# Urgent start peritoneal dialysis: are we there yet?

**DOI:** 10.1186/s12882-020-1706-2

**Published:** 2020-01-31

**Authors:** Keiko I. Greenberg, Bernard G. Jaar

**Affiliations:** 1 Division of Nephrology, Department of Medicine, Johns Hopkins University School of Medicine, Baltimore, MD USA; 2 Department of Epidemiology, Johns Hopkins Bloomberg School of Public Health, Baltimore, MD USA; 3 Welch Center for Prevention, Epidemiology and Clinical Research, Johns Hopkins University, Baltimore, MD USA; 4 Nephrology Center of Maryland, 5601 Loch Raven Boulevard, Suite 3 North, Baltimore, MD 21239 USA

**Keywords:** Peritoneal dialysis, Urgent start, Peritonitis, Technique failure, Complications

## Abstract

The use of peritoneal dialysis (PD) has increased substantially in the United States (US) in the past decade. This was likely spurred in large part by the implementation of the expanded prospective payment system for the Medicare End Stage Renal Disease (ESRD) program in 2011. Over the same period, there has also been growing interest in urgent start PD, which is commonly defined as initiation of PD within 14 days of catheter insertion. Ye and colleagues recently reported their experience with urgent start PD in 2059 Chinese ESRD patients over a 9-year period. Rates of complications, including peri-catheter leaks and peritonitis, were very low despite initiation of PD immediately after open catheter placement via open laparotomy in nearly all patients. Long term technique survival was good, with only 75 patients developing catheter failure. This study provides further evidence to suggest that urgent start PD is feasible and effective, although the generalizability of these results to Western populations is unclear. Recent proposed changes to the payment models in the Medicare ESRD program, designed to incentivize use of kidney transplantation and home dialysis, are likely to further propel growth of PD and urgent start PD in the US. Further studies are needed to optimize use of urgent PD and patient outcomes.

## Background

Peritoneal dialysis (PD) remains underutilized in the United States (US) and many other countries [1]. Despite a substantial increase in the use of PD during the past decade in the US, only 10% of patients with end-stage renal disease (ESRD) on dialysis were using PD at the end of 2016 [2]. Several factors have likely played a role in the predominance of hemodialysis (HD) over PD [3]. HD catheter placement is a routine procedure, while there are fewer experienced physicians willing to place PD catheters (and even fewer available to place PD catheters on short notice) [4]. Outpatient HD placement is fairly straightforward given ample in-center HD capacity, while many PD programs may lack the infrastructure needed to manage an influx of new patients [3]. Further, nephrology training in PD has historically been suboptimal – in a survey of adult nephrology trainees, the vast majority felt “well trained and competent” in acute HD and in-center HD, but only about 30% felt the same about acute PD, and 55% about chronic PD [5]. Not least in importance are the financial incentives which have historically favored in-center HD.

More recently, however, there has been increasing focus on Medicare expenditures for ESRD. In 2011, the expanded prospective payment system for the Medicare ESRD program was implemented – this likely spurred the growth seen in home dialysis modalities, particularly PD, over the past several years [6]. It also appears to have increased interest in urgent start PD, which is usually defined as initiation of PD within 14 days of catheter insertion [4]. This is supported by a recent review of the literature examining 33 studies on the topic, the vast majority of which were published in 2012 or later [7]. The July 2019 announcement of the “Advancing American Kidney Health” initiative by the US Department of Health and Human Services will likely further propel interest in home dialysis modalities including PD. This initiative aims to reduce the development of ESRD, and increase home dialysis and kidney transplant [8]. Specifically, it targeted a very lofty goal of home dialysis or transplant for 80% of new ESRD patients by 2025. A proposed payment model was released which incentivizes ESRD prevention, home dialysis and transplant.

## Main text

It is in this context that we examine the recent article by Ye et al. describing their experience with urgent-start PD over 9 years in China [9]. This was a retrospective cohort study of 2059 ESRD patients who received urgent-start PD between January 1, 2006 and December 31, 2014 at a single institution. Urgent-start PD was defined as “starting PD within 14 days after catheter insertion,” but almost all patients were initiated immediately after catheter placement. Nephrologists placed catheters via open laparotomy. Intermittent PD was prescribed with 500 mL dialysate volume, dwell time 1 h for 8 cycles on day of catheter placement, then 650 ml volume for 1 h for 9 cycles for the next 1–2 days, followed by gradual increase in dialysate volume to 2 L or maximum tolerated volume over the next week. Patients were transitioned to continuous ambulatory peritoneal dialysis (CAPD) approximately 8–10 days after catheter insertion. Within 5–7 days of catheter insertion, patients and caregivers received standardized training. Complications were uncommon, with lower rates of peri-catheter leak and peritonitis seen than in many other studies [7]. Three patients (0.1%) had significant bleeding complicating catheter placement. Within 2 weeks of catheter placement, 24 patients developed peritonitis (0.28 per patient-year) and 7 patients developed exit site infections (0.08 per patient year). Within the first month after catheter placement, 36 patients developed abdominal wall complications – most common was peri-catheter leakage which occurred in 19 patients (0.9%). After the first month, an additional 111 patients developed abdominal wall complications – hernias were the most common, occurring in 70 patients. Median follow up was 36.5 months.

The authors focused on catheter failure as the primary outcome, which was defined as “functional catheter problems that required catheter manipulation or replacement, or lead to technique failure.” Functional catheter problems included any difficulty with instillation or drainage of dialysate for which surgical intervention potentially may have been needed. Functional catheter problems occurred in 156 (7.6%) patients, with 28.2% of these occurring within 7 days of catheter placement, 12.2% between 8 and 14 days, 14.1% between 15 days to 1 month, and 32.1% between 1 month and 1 year. Conservative measures resolved functional catheter problems in 81 (51.9%) patients; the remaining 75 patients had catheter failure. Catheter failure was caused by catheter shift in 65.2% and omental wrapping in 32.0%. In a multivariate model, younger age was independently associated with a higher risk of catheter failure, with a 19% decrease in risk for every 5 year increase in age. Omental wrapping caused significantly more catheter malfunctions in patients ≤50 years old compared to those > 50 years old. Catheter patency rates were 97.6% at 1 month, 96.4% at 1 year, and 96.2% at 3 years and 5 years. During follow up, 291 (14.1%) were transitioned to HD, 430 (20.9%) received a kidney transplant, 534 (25.9%) died and 738 (36.8%) remained on PD. Technique survival rates were 99.5% at 1 month, 97.0% at 1 year, 90.3% at the end of 3 years, and 82.7% at the end of 5 years.

This large cohort study appears to confirm that urgent start PD is a safe and effective dialysis procedure. Limitations of this study included the single center, retrospective design and the lack of a control group such as planned PD. Whether the results can be generalized to Western populations or healthcare systems is unclear. As noted by the authors, in most rural areas of China, PD is the only dialysis modality available. Catheters were placed by nephrologists and not interventional radiologists or surgeons as in the US. The average body mass index of patients included in the study was 21.5 kg/m^2^. Only 21.8% of patients had ESRD due to diabetic nephropathy – diabetic nephropathy was not associated with functional catheter problems or catheter failure, but it was associated with a 56% increase in risk of abdominal wall complications. Although this finding will need to be confirmed in other cohorts, it suggests that more abdominal wall complications may be seen in populations with higher rates of diabetic nephropathy after PD catheter placement by open laparotomy.

Increasing interest in urgent start PD and the recently proposed Medicare payment model are likely to lead to further growth in PD use in the US. Approximately 80% of patients used a catheter at HD initiation in 2016, and the majority of these patients did not have a maturing AV fistula [2]. Using urgent start PD in appropriate patients who are interested in PD could reduce the risks and the costs associated with HD catheters – a 2014 study showed that urgent start PD was less costly than urgent start HD in the first 90 days of unplanned dialysis [10]. Urgent start PD, however, requires dedicated infrastructure and effective protocols. An urgent start PD program would include, at a minimum, the following: objective methods for patient selection, processes for urgent PD catheter placement, hospital support to enable urgent PD initiation in the inpatient setting as needed, nursing support to manage intermittent PD in the outpatient setting, and dialysis unit administrative support to ensure appropriate resources [11]. Establishment of such a program is not an easy task, but likely to have a significant impact on home dialysis as we strive to meet the recently announced goals. Urgent start PD programs would likely increase trainee exposure to PD, meeting an unmet and crucial need in nephrology education, at least in the US [12].

## Conclusions

Despite its limitations, the recent study by Ye et al. adds to a growing body of literature suggesting that urgent start PD is feasible and effective. Recently proposed changes to dialysis reimbursement in the United States are likely to lead to further growth in home dialysis modalities, particularly PD – increased use of urgent start PD may play a critical role in this effort. Certainly, further studies are warranted to optimize its use and patient outcomes.

## Data Availability

Not applicable.

## Abbreviations

CAPD: Continuous ambulatory peritoneal dialysis
ESRD: End-stage renal disease
HD: Hemodialysis
PD: Peritoneal dialysis

## References

[1] Briggs V, Davies S, Wilkie M (2019). International variations in peritoneal dialysis utilization and implications for practice. Am J Kidney Dis.

[2] United States Renal Data System (2018). 2018 USRDS annual data report: Epidemiology of kidney disease in the United States.

[3] Ghaffari A, Kalantar-Zadeh K, Lee J, Maddux F, Moran J, Nissenson A (2013). PD first: peritoneal dialysis as the default transition to dialysis therapy. Semin Dial.

[4] Blake PG, Jain AK (2018). Urgent Start Peritoneal Dialysis: Defining What It Is and Why It Matters. Clin J Am Soc Nephrol.

[5] Berns JS (2010). A survey-based evaluation of self-perceived competency after nephrology fellowship training. Clin J Am Soc Nephrol.

[6] Rivara MB, Mehrotra R (2014). The changing landscape of home dialysis in the United States. Curr Opin Nephrol Hypertens.

[7] Tunbridge M, Cho Y, Johnson DW (2019). Urgent-start peritoneal dialysis: is it ready for prime time?. Curr Opin Nephrol Hypertens.

[8] US Department of Health and Human Services. Advancing American Kidney Health. https://aspe.hhs.gov/system/files/pdf/262046/AdvancingAmericanKidneyHealth.pdf. Accessed 14 Aug 2019.

[9] Ye H, Yang X, Yi C, Guo Q, Li Y, Yang Q (2019). Urgent-start peritoneal dialysis for patients with end stage renal disease: a 10-year retrospective study. BMC Nephrol.

[10] Liu FX, Ghaffari A, Dhatt H, Kumar V, Balsera C, Wallace E (2014). Economic evaluation of urgent-start peritoneal dialysis versus urgent-start hemodialysis in the United States. Medicine (Baltimore).

[11] Ghaffari A, Kumar V, Guest S (2013). Infrastructure requirements for an urgent-start peritoneal dialysis program. Perit Dial Int.

[12] Rope RW, Pivert KA, Parker MG, Sozio SM, Merell SB (2017). Education in nephrology fellowship: a survey-based needs assessment. J Am Soc Nephrol.

